# Mapping the conductivity of graphene with Electrical Resistance Tomography

**DOI:** 10.1038/s41598-019-46713-8

**Published:** 2019-07-23

**Authors:** Alessandro Cultrera, Danilo Serazio, Amaia Zurutuza, Alba Centeno, Oihana Txoperena, David Etayo, Alvaro Cordon, Albert Redo-Sanchez, Israel Arnedo, Massimo Ortolano, Luca Callegaro

**Affiliations:** 1 INRIM — Istituto Nazionale di Ricerca Metrologica, Torino, 10135 Italy; 2 Graphenea, San Sebastián, 20009 Spain; 3 das-Nano, Tajonar, 31192 Spain; 4 Universidad Pública de Navarra, Campus Arrosadía, Pamplona, 31006 Spain; 5 Politecnico di Torino, Torino, 10129 Italy

**Keywords:** Electrical and electronic engineering, Imaging techniques, Electronic properties and devices

## Abstract

Electronic applications of large-area graphene films require rapid and accurate methods to map their electrical properties. Here we present the first electrical resistance tomography (ERT) measurements on large-area graphene samples, obtained with a dedicated measurement setup and reconstruction software. The outcome of an ERT measurement is a map of the graphene electrical conductivity. The same setup allows to perform van der Pauw (vdP) measurements of the average conductivity. We characterised the electrical conductivity of chemical-vapour deposited graphene samples by performing ERT, vdP and scanning terahertz time-domain spectroscopy (TDS), the last one by means of a commercial instrument. The measurement results are compared and discussed, showing the potential of ERT as an accurate and reliable technique for the electrical characterization of graphene samples.

## Introduction

The electrical properties of graphene can be assessed with different techniques. Contact methods, like the inline four-point probe (4PP) and the van der Pauw (vdP) methods, achieve the highest measurement accuracy. The 4PP method even allows for some spatial resolution, but it is destructive on graphene^1–3^. Conversely, if supported by appropriate models, contactless scanning methods, like terahertz time-domain spectroscopy (TDS) and Raman spectroscopy, can provide indirect measurements of conductivity with higher spatial resolution than the 4PP method.

A way to perform accurate and spatially resolved electrical measurements is the *electrical resistance tomography* (ERT). This technique generates a map of the electrical conductivity of the interior of a two- or three-dimensional sample from a set of four-terminal resistance measurements performed at its boundary^4–6^. Applications of ERT and, more generally, of its equivalent in the ac regime, the *electrical impedance tomography* (EIT), are found in many research and industrial fields. For example, in civil engineering, ERT/EIT can be applied to multi-phase flow detection and measurement^7,8^. In geology, the technique can be applied to the exploration of soils and aquifers^9,10^. Medical applications of EIT, the most studied, include real-time visual monitoring of breathing and cardiac activity^6^. A system for the recognition of wrist gestures has been recently reported^11^.

ERT/EIT implementations with fast multiplexers and analogue-to-digital converters achieve millisecond measurement times, thus allowing real-time imaging with reconstruction rates of the order of 100 Hz^8,12,13^. For these applications, the goal is to get high image contrast, to highlight inhomogeneities and to record the temporal evolution of the samples rather than to accurately measure conductivity values. However, since ERT can be considered as a kind of highly-redundant vdP method, accurate quantitative measurements should also be possible: in fact, the authors have recently shown that ERT can provide accurate conductivity maps of thin film samples^14^.

The basis of ERT is the solution of a so-called inverse problem: the two- or three-dimensional conductivity map is obtained from a discrete set of four-terminal resistance measurements. The solution of the inverse problem requires regularisation techniques and dedicated numerical methods: for this purpose, both commercial^15^ and open source^16^ ERT packages are available.

In the following, we describe a setup, based on commercial electrical instruments and a dedicated measurement fixture, for the measurement of ERT maps on large-area graphene samples. Measurements are performed on large-area graphene samples on an insulating substrate. vdP and TDS measurements are also performed on the same samples and the results are compared.

## Methods

### ERT setup

The input quantities of the ERT method are a set of four-terminal resistances. Their measurement is performed by contacting the sample at its boundary.

**Figure 1:**
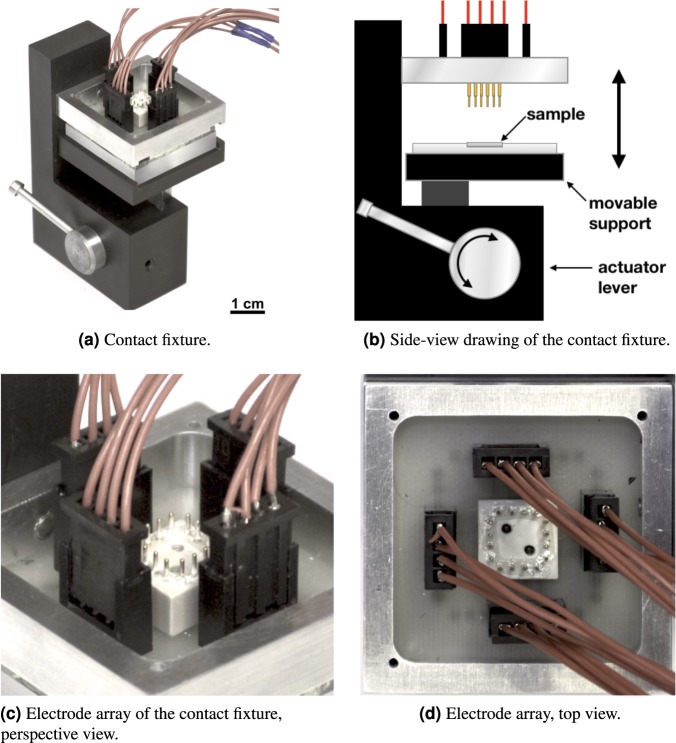
(**a**) Photograph of the contact fixture. (**b**) Side-view drawing of the fixture. (**c**,**d**) Details of the 16 spring-loaded electrodes. The fixture accepts 1 × 1 cm samples. A sample is first loaded on a milled plastic support and then the actuator lever is operated to lift the sample until it makes contact with the spring-loaded needles. The electrodes touch the sample at 500 μm from its edges. The diameter of each needle tip is 40 μm. The vertical-rail loading mechanism limits the applied force to 0.15 N.

A dedicated measurement setup has been implemented. The sample is contacted with *n* = 16 electrodes using a custom fixture, as shown in Fig. 1.

**Figure 2:**
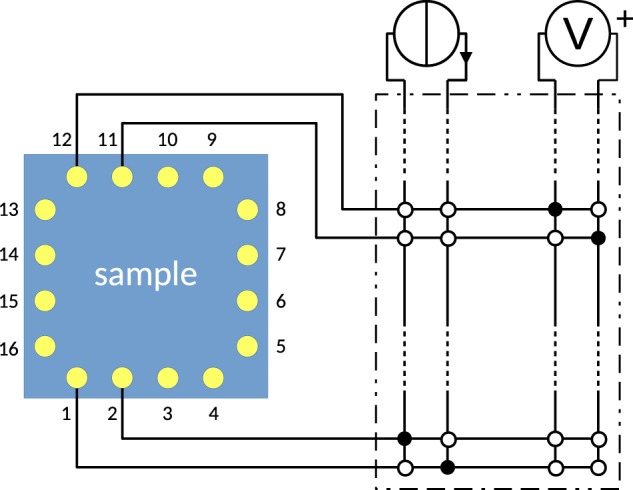
Electrical schematic diagram of the measurement setup. The 16 discs inside the sample area represent the spring-loaded electrodes of Fig. 1. The distance between nearby contacts along an edge is 2 mm, while the distance between nearby contacts at a corner is 2.12 mm. The dash-dotted box represents the switching unit, which includes 4 × 8 relay switches (only a few are drawn). A full (empty) dot represents a closed (open) relay switch. In the present setup, the dc voltmeter is a Keysight 34461A; the dc ammeter is a Keithley K2602B; the switching unit is a Keysight 34933A reed relay matrix in a 34480A host. All instruments are interfaced through an IEEE-488 bus.

The four-terminal resistance measurements are performed with the circuit of Fig. 2, consisting of a current source and a voltmeter connected to the electrode array through a relay scanner.

To perform an ERT measurement, a measurement pattern should be defined. A measurement pattern is the sequence of current injecting contact pairs and voltage measurement pairs used to perform the four-terminal resistance measurements. Several different measurement patterns can be considered^17–19^. There are two important aspects that need to be taken into account when choosing a measurement pattern: (i) the spatial resolution depends on the number of resistance measurements generated by the pattern; (ii) the accuracy of the reconstructed conductivity map depends on the accuracy associated to the resistance measurements defined by the pattern. In general, there is a trade-off between these two aspects because patterns that generate a large number of measurements are more affected by the measurement noise^19^.

In our implementation, we employed an *adjacent* pattern: the current *I* is injected in a pair of adjacent contacts (e.g., 1–2, with contact *n* considered adjacent to contact 1) and the voltage *V* is measured across all remaining pairs of adjacent contacts (3–4, 4–5, …); then, the current is switched to the next adjacent pair (e.g., 2–3) and the voltage measurement is repeated across all other pairs. In total, a set of *n*(*n* − 3) different measurements is obtained.

The current was set to 100 μA to get voltages of the order of tens of microvolt, a level suitable to be measured with high accuracy with the present setup. To check whether this current level was acceptable, a series of measurements with currents ranging from 10 μA to 100 μA was performed. The compatibility of the resistance values obtained at different current levels excluded self-heating effects on the samples, and confirmed the ohmic nature of the contacts. To minimise offset and noise, each measurement was performed at currents ±*I* and averaged over 10 repetitions. The total measurement time was about 400 s. The measurements were performed in a shielded and temperature-controlled environment at (23.0 ± 0.5) °C.

All instruments were calibrated on the measurement ranges employed during the experiments. Instrument specifications and measurement repeatability allow to evaluate^20^ the combined (type A + B) uncertainty of the four-terminal resistance measurements. The resulting relative uncertainty is less than 4 × 10^−3^. In absence of applied magnetic field, the sample can be considered as an *n*-terminal reciprocal network, and a validation of the measurements is given by the reciprocity error, the difference between two reciprocal transresistance measurements. Here the relative average reciprocity error is less than 1 × 10^−3^.

The measurement setup allows also to perform automated vdP measurements over a large number of vdP contact configurations.

### ERT image reconstruction

Consider a conductive region Ω bounded by the surface Σ. Let *σ*(*P*) be the conductivity at each point *P* of Ω. The electrostatic problem^21^ is governed by the Laplace equation

1 $$\nabla \cdot [\sigma (P)\nabla \varphi (P)]=0,$$

where ∇ and ∇⋅ are, respectively, the gradient and the divergence operators, and *ϕ*(*P*) is the electrostatic potential.

The *forward* electrostatic problem consists in determining *ϕ*(*P*) when *σ*(*P*) is known at each point *P* of Ω and a set of electrical boundary conditions are given at the surface Σ. The solution to this problem yields also the current density ***J***(*P*) = −*σ*(*P*)∇*ϕ*(*P*). The boundary conditions of the ERT forward problem involve *n* points on the conductor surface Σ, which we can interpret as *n* contacts, to which terminals can be connected. The conductor can be thus considered as an *n*-terminal passive electrical network ${\mathscr{N}}$. If the currents *I*_*k*_, *k* = 1, …, *n*, through terminals are given, it can be shown^22^ that the ERT forward problem has a unique solution *ϕ*(*P*) in the volume Ω (up to the choice of a terminal reference potential).

The *inverse* electrostatic problem consists instead in determining *σ*(*P*) in Ω from information on *ϕ*(*P*) and ***J***(*P*) at the surface Σ. In the ERT inverse problem, the information is given in the form of a set ***R*** of four-terminal resistances of ${\mathscr{N}}$, *R*_*pq*,*rs*_ = *V*_*rs*_/*I*_*pq*_, with *p*, *q*, *r*, *s* = 1, …, *n*, and where *V*_*rs*_ is the open-circuit voltage across terminals *r* and *s* when current *I*_*pq*_ flows from terminal *p* to terminal *q* with all the other terminals left open. Since the measurement of *V*_*rs*_ samples *ϕ*(*P*) only at discrete points of the surface Σ, the given input information is incomplete and the ERT inverse problem is ill-posed, ill-conditioned and nonlinear^23^. The ill-posed nature of the ERT inverse problem requires a regularisation technique^6,24,25^. Detailed formulations of the ERT problem, and thorough discussions about the existence and uniqueness of a solution, are given in the bibliography^5,6,26,27^.

The ERT map reconstruction here employed is based on the Tikhonov functional^28^ [section 5]

2 $$\hat{{\boldsymbol{\sigma }}}={\rm{\arg }}\mathop{{\rm{\min }}}\limits_{{\varsigma }}||{{\boldsymbol{R}}}^{F}({\boldsymbol{\varsigma }})-{\boldsymbol{R}}{||}^{2}+{\lambda }^{2} {\mathcal R} ({\boldsymbol{\varsigma }}),$$

where $\hat{{\boldsymbol{\sigma }}}$ is the estimate of the conductivity map *σ*(*P*); ***ς*** is the (map) argument of the functional, ||***R***^*F*^(***ς***) − ***R***|| is the norm of the deviation of ***R***^*F*^(***ς***), the solution of the forward problem for ***ς***, from ***R***. The last term, ${\mathcal R} ({\boldsymbol{\varsigma }})$, is the regularisation term and the scalar *λ* is the regularization parameter. ERT problems are typically solved by finite-element methods^29,30^.

We developed an ERT image reconstruction code based on EIDORS (Electrical Impedance and Diffuse Optical tomography Reconstruction Software), an open source package of functions running on MATLAB and Octave platforms^31^. The code generates a two-dimensional finite-element square mesh (6994 elements) having the same size of the sample, with 16 contacts placed as in the experimental fixture. A point contact model was considered appropriate because of the small size of the physical contacts compared to the mesh element size^32^. The ERT solution $\hat{{\boldsymbol{\sigma }}}$ is obtained with an absolute iterative reconstruction approach based on the Gauss-Newton solver available in EIDORS. We employed Tikhonov regularisation with Laplace prior constraint^33^. The strategy employed for the selection of the regularisation parameter *λ* is the L-curve method^34^.

The finite element solution $\hat{{\boldsymbol{\sigma }}}$ is interpolated and rediscretised (with EIDORS routines) on a grid of the same size as the TDS maps (100 × 100 pixel) to make these directly comparable. The image resolution depends primarily on the amount of available boundary measurements and it is thus limited by the number of contacts. In practice, features smaller than the inter-electrode distance are smeared out^14,35^. Other factors that can affect the image resolution are the mesh density and the amount of regularisation. In our experiments the mesh was dense enough to have no substantial influence on the spatial resolution.

The expression of uncertainty of the conductivity values of an ERT map is an open problem, in particular for the determination of the sensitivity with respect to the input data (the four-terminal resistance measurements) and the specific reconstruction method chosen. Preliminary numerical simulations suggest to assign to the maps presented below a conservative relative uncertainty of a few percent.

**Figure 3:**
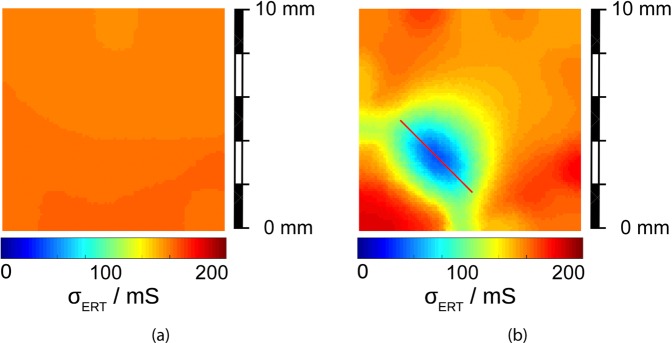
Test of the ERT method. Conductivity maps of a FTO sample were measured (**a**) before and (**b**) after performing a thin straight cut (red line).

A test measurement on a fluorinated tin oxide (FTO) thin film is reported in Fig. 3. The sample, of uniform conductivity, was measured before (Fig. 3a) and after (Fig. 3b) the surface had been damaged with a thin linear cut. The conductivity values of Fig. 3a have a relative standard deviation of 1.6%; the conductivity dip in Fig. 3b has a full width at half maximum (measured over the sample diagonal) of 2.69 mm, to be compared with the average distance between two adjacent contacts of 2 mm. Other test measurements on the same material, with different geometries, are reported in a previous work^14^.

### Time domain terahertz spectroscopy

In terahertz time-domain spectroscopy, an electromagnetic sub-picosecond pulse is focussed on the sample. The amplitude of the reflected component (*THz waveform*) is measured in the time domain. The Fourier transform of the waveform gives the frequency components of the pulse, which typically span the range from 100 GHz to 5 THz^36–43^.

The measurements were performed with a Onyx TDS scanner, a graphene and 2D material quality inspector developed by das-Nano^44^. A sketch of the system is reported in Fig. 1b of Ladrón *et al.*^45^. A fibre-coupled laser generates femtosecond optical pulses from which pulses are generated and detected by conversion with photoconductive antennas^40–42^. A pump-and-probe scheme allows then to measure the time evolution of the THz waveform. An example of detected signal is shown in Fig. 1d of Ladrón *et al.*^45^. The optical path of the beam includes focussing polyethylene lenses. A mechanical scanning system controls the position of the beam on the sample^46^. The ac conductivity is determined from the Fourier transform of the THz waveform (see Eq. 1 in Buron *et al.*^47^). Fitting the Drude-Smith model^48^ to the ac conductivity (see Fig. 2 in Buron *et al.*^49^) allows to recover the dc conductivity of the sample.

For the reported measurements, the system was configured in normal reflection geometry (emitter and detector located on the same side of the sample) with a 25 mm focal length and a bandwidth of up to 5 THz. The broadband pulse includes frequencies from 0.1 THz to 3 THz, yielding an average spatial resolution of 600 μm. However while the stronger beam component is around 0.5 THz, information is also present at the higher frequency that corresponds to a spatial resolution of 100 μm. Hence, a scanning resolution (pixel size) of 100 μm was chosen to avoid any loss of information and possible image aliasing. On 1 × 1 cm samples, this produced 100 × 100 pixel maps.

### Sample preparation

The monolayer graphene samples were grown by the authors on a 18 μm-thick copper foil catalyst surface inside a 4-inch chemical vapour deposition (CVD) reactor AIXTRON BM. Before the graphene growth, the copper foil is annealed for 15 min at 1000 °C using a mixture of argon and hydrogen to reduce the native copper oxide and increase the grain size. The graphene growth is performed at 1000 °C for 10 min using a mixture of methane and hydrogen in 1:4 ratio with argon as gas carrier. After the growth phase, the system is cooled down to room temperature under a hydrogen and argon atmosphere. A poly-methyl methacrylate (PMMA) coating is spun on the grown graphene and the copper foil is then etched in an aqueous solution of FeCl_3_. After the etching, the FeCl_3_ is removed by rinsing the sample in several baths of deionised water. Finally, the PMMA/graphene stack is transferred onto a quartz substrate wafer and the PMMA is removed in acetone. The wafer is cut into 1 × 1 cm chips. All measurements are performed after cutting.

The test sample of Fig. 3 is a commercial fluorine-doped tin-oxide film (FTO; SnO_2_:F) on a glass substrate. FTO is highly doped and its electrical behaviour is metallic^14^. A laser scriber was used to define the sample geometry and to cut the defect line of Fig. 3b, which has a length of 4.5 mm and a thickness of about 200 μm.

## Results and Discussion

### Conductivity maps

The electrical conductivity of the CVD graphene samples was measured with the methods ERT, TDS and vdP. ERT and TDS returned maps of local conductivity values, *σ*_ERT_ and *σ*_TDS_. For each sample, the averages of these maps were computed over the whole sample (${\sigma }_{{\rm{ERT}}}^{\square }$, ${\sigma }_{{\rm{TDS}}}^{\square }$) and over a square area of 3.5 × 3.5 mm around the sample centre (, ). The conductivity measured with the vdP method is labelled as *σ*_vdP_. For clarity, the symbols are listed in Table 1.

**Table 1:** Symbols used in the text.

Symbol	Meaning
*σ*_ERT_	Local conductivity by ERT
*σ*_TDS_	Local conductivity by TDS
${\sigma }_{{\rm{ERT}}}^{\square }$	Average of *σ*_ERT_ over the whole sample area
${\sigma }_{{\rm{ERT}}}^{\square }$	Average of *σ*_TDS_over the whole sample area
	Average of *σ*_ERT_ over an area of $3.5\times 3.5$ around the sample centre
	Average of *σ*_TDS_ of $3.5\times 3.5$ around the sample centre
*σ*_vdP_	Conductivity by vdP

**Figure 4:**
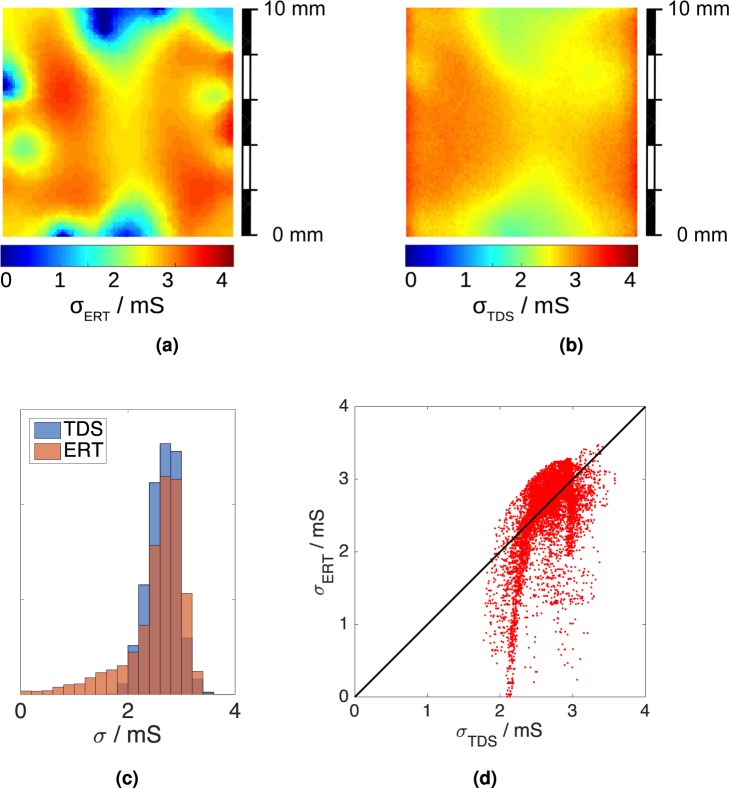
Sample S40: (**a**) ERT conductivity map and (**b**) TDS map. The maps have 100 × 100 pixels and the color scale represents the conductivity in. (**c**) ERT and TDS conductivity distributions. (**d**) Pixel-to-pixel scatter plot of ERT versus TDS conductivity values. Each dot has coordinates (*σ*_ERT_, *σ*_TDS_) and the solid line is the quadrant bisector, for which *σ*_ERT_ = *σ*_TDS_.

**Figure 5:**
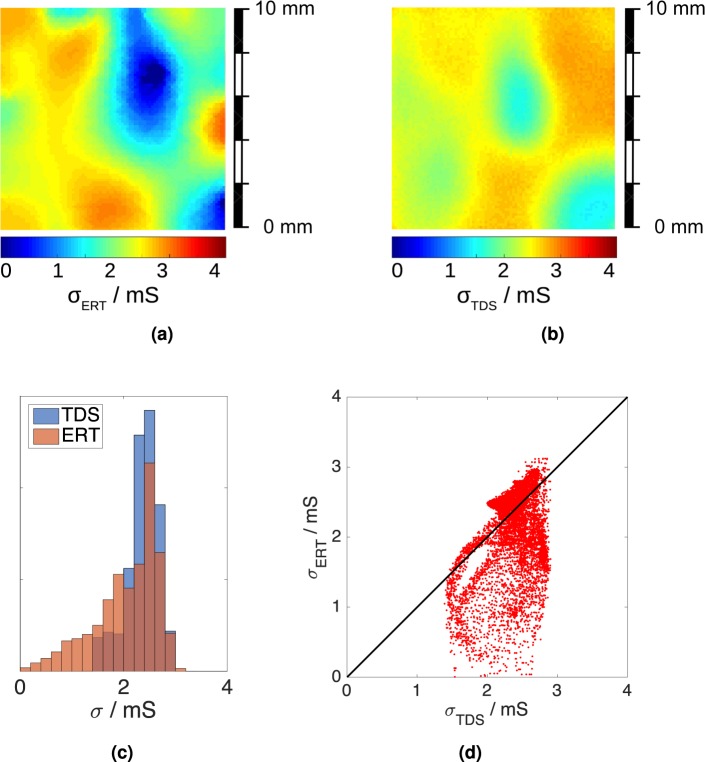
Sample S28: (**a**) ERT conductivity map and (**b**) TDS map; the maps have 100 × 100 pixels and the color scale indicates the conductivity in. (**c**) the ERT and TDS conductivity distributions. (**d**) the pixel-to-pixel scatter plot of ERT versus TDS conductivity values. Each dot has coordinates (*σ*_ERT_, *σ*_TDS_); the solid line is the quadrant bisector, for which *σ*_ERT_ = *σ*_TDS_.

Figures 4 and 5 show the results of the measurements on the graphene samples labelled S40 and S28. Each figure shows the ERT map, the TDS map, the ERT and TDS conductivity distributions, and a scatter plot of the ERT conductivity versus the TDS conductivity for each pixel.

Both ERT and TDS maps show significant conductivity variations across the samples. Similar variations were reported in other works^49–51^ and can be related to the quality of the growth process (which affects the grain size^52,53^), to the presence of bi- or multi-layer seeds^54^, to the quality of the transfer process (which can cause wrinkles or cracks^55,56^ and leave polymer residues^57^) and to local adsorption of environmental contaminants. The average conductivity values reported in Table 2 range from 1.5 mS to 2.9 mS, a range consistent with the literature on large area CVD graphene^56,58–61^. ERT maps appear more contrasted than TDS ones. Comparisons of the conductivity distributions (Figs 4c and 5c) and of the ERT-TDS pixel scatter plots (Figs 4d and 5d) show that the ERT maps extend to lower conductivity values than the TDS maps. The discrepancy between ERT and TDS maps could be explained by a different sensitivity of the two techniques to line defects of zero conductivity, such as tears, having a width much below the resolution. TDS performs an average over the spot size, whereas ERT is based on global electric current flow paths which are diverted by line defects. Such an effect in TDS maps has been previously reported in the literature^49^ [Fig. 3c].

**Table 2:** Electrical conductivity obtained with ERT, TDS and vdP methods on samples S40 and S28.

Sample	${\sigma }_{{\rm{ERT}}}^{\square }$		${\sigma }_{{\rm{TDS}}}^{\square }$		*σ*_vdP_
S40	2.531	2.669	2.663	2.565	2.880 [2.38–3.23]
S28	2.050	1.323	2.214	2.878	1.631 [1.16–2.98]

Symbols are defined in Table 1. The vdP value *σ*_vdP_ is measured choosing contacts close to the four corners; the interval includes the values measured with the other electrode configurations. All values are expressed in mS.

The ERT map of S40 is shown in Fig. 4a. *σ*_ERT_ is lower at the top and bottom edges of the sample, and substantially uniform in its interior. The same features can be recognised also in the TDS map of Fig. 4b. ${\sigma }_{{\rm{ERT}}}^{\square }$ and ${\sigma }_{{\rm{TDS}}}^{\square }$ are in reasonable agreement with a relative difference of 5.1%. If we consider the averages  (S40) and  (S40) around the sample centre, where the maps have the best visual match, the agreement slightly improves to 4%. The lower conductivity spots on the sample edge are likely to be due to dicing/transfer defects having zero conductivity. The distribution of *σ*_ERT_ (Fig. 4c) actually reaches a zero value, whereas the range of *σ*_TDS_ has a narrower span, possibly related to the broader resolution of the TDS method. Concerning S28, the ERT and TDS maps are shown respectively in Fig. 5a,b. The main feature is the presence of two low conductivity spots, one near the sample centre, and one at the bottom right corner. This can be clearly recognised in both the ERT and TDS maps. The discrepancy between ${\sigma }_{{\rm{ERT}}}^{\square }$ (S28) and ${\sigma }_{{\rm{TDS}}}^{\square }$ (S28) is 7.7%, larger than that obtained for S40. This is reflected in the longer tail in the distribution of *σ*_ERT_ (Fig. 5c) for low conductivity values, compared to that of *σ*_TDS_.

### van der Pauw measurements

Table 2 reports the *σ*_vdP_ conductivity measured in the typical configuration, using contacts close to the sample corners. The measurement system, however, allows for many other configurations and the interval of values covered by these measurements is also reported. The vdP method^2^ requires samples of homogeneous conductivity (for this ideal case all the measurements in the different contact configurations would give the same value). The spatial sensitivity function of the vdP method has been derived for the square geometry, with contacts positioned at the corners or at the edges^62^, showing that the vdP method is highly sensitive to the conductivity in the central region, and that the sensitivity goes to zero at the sample border. The vdP conductivity *σ*_vdP_ (S40) is 13.8% greater than ${\sigma }_{{\rm{ERT}}}^{\square }$ (S40) and 7.9% than  (S40). This is consistent with the ERT map (Fig. 4a), where *σ*_ERT_ around the sample centre is greater than that at the border. In sample S28, *σ*_vdP_ (S28) is 20.4% less than  (S28) but 18.8% greater than  (S28), because S28 has a conductivity dip in the sample centre. The mismatch between the vdP and the ERT measurements can be related to the conductivity inhomogeneities that strongly affect the vdP results. The large intervals given for the vdP measurement in Table 2 are an independent evidence of these inhomogeneities. Note that the reported intervals of the vdP measurements include the values ${\sigma }_{{\rm{ERT}}}^{\square }$ and . An additional contribution to the mismatch between vdP and the other techniques is due to the position of the contacts, not exactly lying along the sample border. This contribution can be estimated of about 1%^63,64^.

## Conclusion

We have shown that electrical resistance tomography can be an accurate and easy-to-implement technique for the measurement of electrical conductivity maps of large area graphene samples. Being a contact method, the conductivity map can be obtained without referring to a physical conductivity model, and the measurement traceability can be achieved by routine calibration of the electrical instruments employed. The effects of the substrate is negligible as long its resistivity is sufficiently high. The application of the ERT technique is not bound to the size of the samples here considered. With proper fixtures, wafer-size measurements are possible, the ERT spatial resolution being set by the inter-electrode distance. High-throughput, real-time measurements can also be envisaged.

## Data Availability

The data sets generated during and/or analysed during the current study are available from the corresponding author on reasonable request.
